# Exploring the role of genetic variations in NAFLD: implications for disease pathogenesis and precision medicine approaches

**DOI:** 10.1186/s40001-024-01708-8

**Published:** 2024-03-20

**Authors:** Seyedeh Kosar Mahmoudi, Shadi Tarzemani, Taha Aghajanzadeh, Mohammadreza Kasravi, Behzad Hatami, Mohammad Reza Zali, Kaveh Baghaei

**Affiliations:** 1 Basic and Molecular Epidemiology of Gastrointestinal Disorders Research Center, Research Institute for Gastroenterology and Liver Diseases, Shahid Beheshti University of Medical Sciences, Tehran, 1985714711 Iran; 2 Gastroenterology and Liver Diseases Research Center, Research Institute for Gastroenterology and Liver Diseases, Shahid Beheshti University of Medical Sciences, Tehran, 1985714711 Iran

**Keywords:** NAFLD, NASH, Gene variants, Lean NAFLD, Polymorphism, Precision medicine

## Abstract

Non-alcoholic fatty liver disease (NAFLD) is one of the leading causes of chronic liver diseases, affecting more than one-quarter of people worldwide. Hepatic steatosis can progress to more severe forms of NAFLD, including NASH and cirrhosis. It also may develop secondary diseases such as diabetes and cardiovascular disease. Genetic and environmental factors regulate NAFLD incidence and progression, making it a complex disease. The contribution of various environmental risk factors, such as type 2 diabetes, obesity, hyperlipidemia, diet, and sedentary lifestyle, to the exacerbation of liver injury is highly understood. Nevertheless, the underlying mechanisms of genetic variations in the NAFLD occurrence or its deterioration still need to be clarified. Hence, understanding the genetic susceptibility to NAFLD is essential for controlling the course of the disease. The current review discusses genetics’ role in the pathological pathways of NAFLD, including lipid and glucose metabolism, insulin resistance, cellular stresses, and immune responses. Additionally, it explains the role of the genetic components in the induction and progression of NAFLD in lean individuals. Finally, it highlights the utility of genetic knowledge in precision medicine for the early diagnosis and treatment of NAFLD patients.

## Background

Non-alcoholic fatty liver disease (NAFLD) is a spectrum ranging from benign simple hepatic steatosis to more advanced forms involving inflammation and fibrosis formation, namely non-alcoholic steatohepatitis (NASH) and cirrhosis, which are also critical predisposing factors in hepatocellular carcinoma (HCC) pathogenesis [1]. This condition is one the most common causes of chronic liver disease [2], afflicting more than one-quarter of the worldwide population [3]. NAFLD burden is progressively increasing and is projected to become the leading cause of liver transplantation. However, no effective therapeutic option for its advanced forms has been found to relieve its cost burden to date [4].

NAFLD incidence and progression depend on various factors, including environmental, genetic, metabolic, and immunologic. Environmental risk factors such as high-fat diets and physical inactivity can develop obesity and type two diabetes mellitus (T2DM) that enhance the accumulation of lipid droplets in hepatocytes. Subsequently, cellular stresses such as oxidative stress could mediate inflammation and fibrosis [5]. However, with similar environmental and metabolic risk factors, a broad inter-individual and inter-ethnic diversity of the phenotypes exists, indicating distinct susceptibility of patients to disease onset and progression [6]. Familial clustering and epidemiological findings suggest a critical role for genetic polymorphisms in determining personalized susceptibility to NAFLD [7]. Various studies demonstrate a higher incidence of liver steatosis and fibrosis in the first-degree relatives of NAFLD probands compared to those of healthy controls [8, 9]. Besides, data related to epidemiological studies on the US population indicate that NAFLD prevalence in African Americans is significantly lower compared to whites and Latinos, who suffer the most [10].

The genetic implication of NAFLD is mainly mediated via single nucleotide polymorphisms (SNPs) in genes contributing to hepatic uptake of fatty acids, lipid droplet biology, very low-density lipoproteins (VLDLs) transportation, de novo lipogenesis (DNL), gluconeogenesis, glycogenolysis, insulin resistance (IR), endoplasmic reticulum stress (ER stress), oxidative stress, autophagy and inflammation [11]. The presence of multiple SNPs associated with NAFLD provides a more comprehensive understanding of the underlying genetic factors contributing to the disease. Furthermore, discussing the effect of different types of mutations on NAFLD can provide valuable insights into the genetic underpinnings of the disease and the identification of preventive and therapeutic strategies [12].

Initial genetic studies were conducted on selective candidate genes. They focused on finding an association between some gain or losing function mutations on the specific genes and NAFLD onset or progress. However, these studies only evaluated limited genes at once. They were unsuccessful in finding new variants or deciphering the probable interplay between different SNPs that affect the course of the disease [13, 14]. Subsequently, genome-wide association studies (GWAS) followed by whole-genome and whole-exome sequencing strategies have significantly improved our understanding of NAFLD hereditability via simultaneous study on millions of SNPs in the genome and a specific phenotype. GWAS, as a population scale study, has uncovered a significant number of variants closely associated with the development of NAFLD in different stages. In these studies, various genes could be simultaneously evaluated to decode the independent association that exists between a genetic variation and the NAFLD [4].

A comprehensive understanding of the polygenic structure of NAFLD is also the prerequisite for risk assessment and developing treatment strategies. Since the genetic signature of each patient is unique, detecting defective genes will show us the affected pathogenic molecular pathways and aid us in early detection, designing effective therapies, and even helping the patients to change their lifestyle in a specific manner [15].

In summary, here we aimed to appraise the contribution of genetics in the pathogenesis of NAFLD. For a better understanding and according to the gene function, we have categorized the genetic polymorphisms regarding their function into lipid and glucose metabolism, cellular stress, and immune system subgroups. Likewise, we discussed the contribution of genetic polymorphism in lean NAFLD. Furthermore, to overcome the inadequacies associated with NAFLD diagnosis and treatment, we discuss the importance of finding these genetic polymorphisms and their potential application in translational medicine to screen genetically predisposed individuals and alleviate the burden of NAFLD by developing precision medicine.

## Metabolic-related genes influencing NAFLD

### Lipid metabolism

**Fig. 1:**
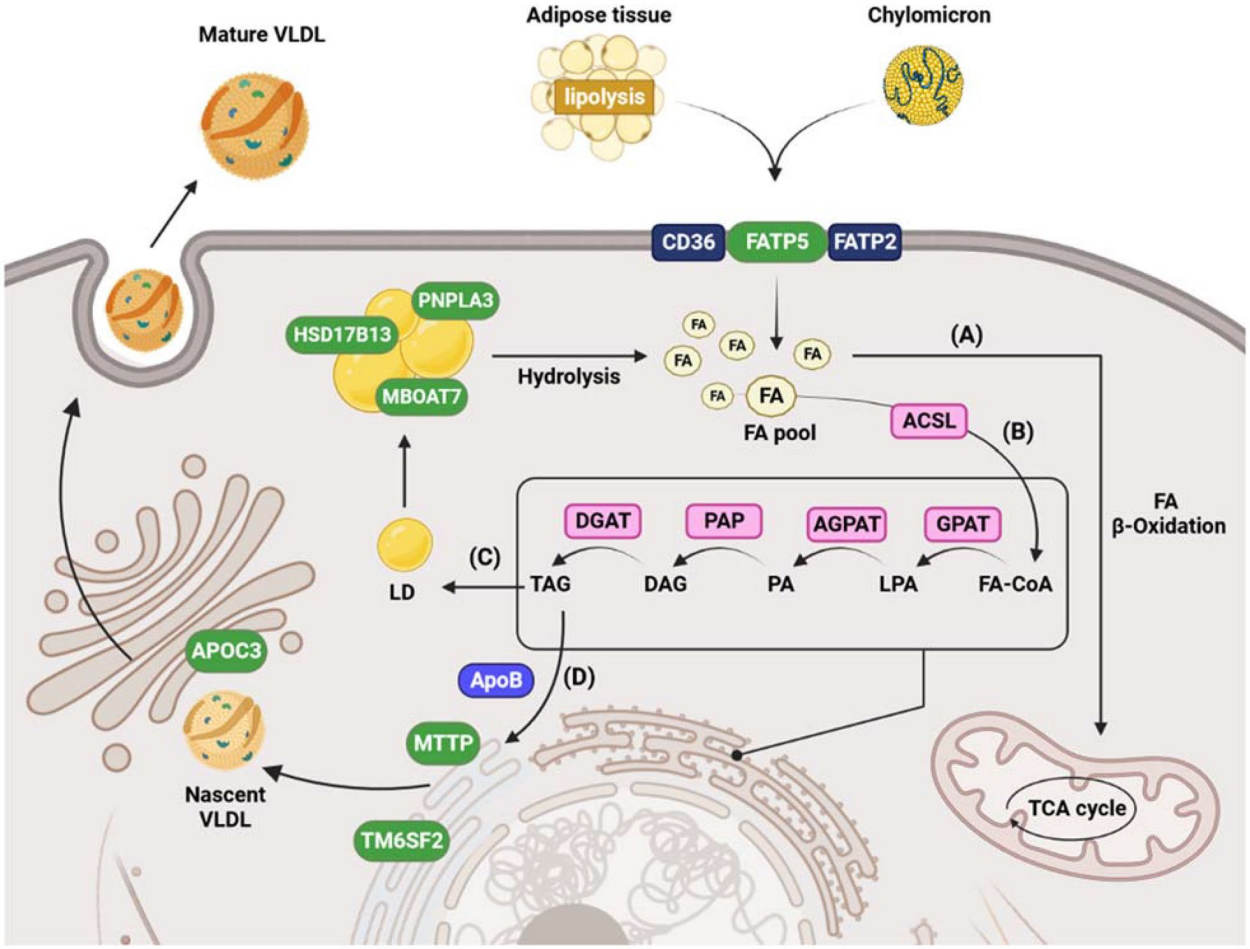
Schematic diagram of lipid metabolism in NAFLD. The uptake of circulating fatty acids from chylomicron remnants or adipose tissue by FATP2/5 and CD36 leads to lipid accumulation in the liver. Fatty acid can take part in several pathways and transferred to the mitochondria and participate in β-oxidation (**A**). ACSL converts fatty acids to FA-CoA, which then enters the TAG synthesis pathway via chain reactions catalyzed by GPAT, AGPAT, PAP, and DGAT (**B**). Produced TAGs can be stored as LDs. PNPLA3, MBOAT7, and HSD17B13 are located on the surface of lipid droplets in hepatocytes. PNPLA3 catalyzes the hydrolysis of TG, MBOAT7 plays an important role during the noncanonical hepatic triglyceride synthesis pathway, and HSD17B13 appears to be involved in hepatic lipid biogenesis and metabolism (**C**). At the ER, TAGs can also be packaged into VLDL by MTTP and ApoB. TM6SF2 is also located in the ER and regulates VLDL secretion. Nascent VLDL particles packaged into VLDL transport vesicles are transported from the ER to the Golgi apparatus, and Apoc3, a component of VLDL, stimulates VLDL assembly and secretion. Finally, mature VLDLs are secreted through vesicle-mediated exocytosis (**D**)

Accumulation of lipid droplets (LDs) in hepatocytes is the initiation point in NAFLD onset, and it is essential to explore the respective metabolic processes. LDs mainly consist of triglycerides (TGs), derived from increased hepatic free fatty acid (FFA) flux due to lipolysis of adipose tissue and de novo lipogenesis in hepatocytes [16]. In the context of increased hepatic FFAs, various gene families control the endoplasmic reticulum function of LD formation [17]. The LDs’ surface contains proteins, which are involved in lipid generation, stabilization, and degradation (Fig. 1) [18, 19]. The most important genes that play a role in lipid metabolism are summarized in Table 1.

**Table 1:** Genetic variants influencing NAFLD pathogenesis via lipid metabolism pathways

Gene	Function	Variant	Mutation type	Variant classification	Population	References
PNPLA3	Triglyceride degradation	rs738409	Missense	Benign	Spanish	[60]
					Brazilian	[23]
		rs139051	Intronic variant	Benign	Han Chinese	[30]
		rs2294918	Missense	Benign	Italian	[29]
		rs6006460	Missense	Likely benign	American	[31]
		rs12483959	Intronic variant	Benign	Korean	[61]
		rs2281135	Intronic variant	Benign	Mexican-American	[62]
					Korean	[61]
					Indian	[63]
MBOAT7	Remodeling of phosphatidylinositol	rs641738	Missense	Uncertain Significance	Italian children	[64]
HSD17B13	Retinol dehydrogenase activity	rs72613567	Donor splice	Benign	Non-Hispanic whites	[36]
LPIN1	Lipogenesis	rs13412852	Intronic variant	Benign	Italian	[65]
ADRB3	Induction of lipolysis	rs4994	Missense	Benign	Japanese	[66]
LYPLAL1	Triglyceride lipase in adipose tissue	rs12137855	Intronic variant	Benign	American	[67]
PPARGC1A	Fatty acid oxidation	rs8192678	Missense	Benign	Han Chinese	[68]
TM6SF2	VLDL secretion	rs58542926	Stop	Benign	Western Chinese	[46]
CETP	Cholesterol metabolism	rs12447924 rs12597002	Nonsense	Benign	Australian	[69]
PEMT	Phosphatidylcholine biosynthesis	rs7946	Missense	Benign	Indian	[70]
MTTP	VLDL secretion	rs1800591	Intronic variant	Benign	Mexican	[71]
APOC3	Inhibitor of lipoprotein lipase activity and triglycerides clearance	rs2854116	Upstream variant	Benign	Chinese	[72]
		rs2070667	Intronic variant			[55]
FATP5	Fatty acid uptake	rs56225452	Upstream variant	Benign	Japanese	[73]

*LPIN1* lipin 1, *ADRB3* adrenoceptor beta 3, *LYPLAL1* lysophospholipase like 1, *PPARGC1A* PPARG coactivator 1 alpha, *CETP* cholesteryl ester transfer protein, *PEMT* phosphatidylethanolamine *N*-methyltransferase

The PNPLA3 gene, the primary gene involved in LD metabolism, is widely expressed in human hepatocytes and hepatic stellate cells (HSCs) and encodes membrane proteins found on the surface of lipid droplets. PNPLA3 protein displays lipase activity toward retinyl esters and triglycerides in hepatocytes and hepatic stellate cells, respectively. The stimulation of lipophagy, which eliminates excess lipids collected in hepatocytes, is caused by PNPLA3’s interaction with LC3-II on the surface of lipid droplets [20, 21]. Genetic alterations in PNPLA3 affect LD protein content and interfere with LD degradation [17, 22]. A substitution mutation at position 148 of this gene results in a loss-of-function protein (I148M) that is strongly associated with increased liver fat content and decreased lipid catabolism [23]. This mutation also exacerbates liver inflammation and increases the risk of NASH disease [24]. Indeed, this genetic variant is associated with steatohepatitis, elevated plasma liver enzymes, liver fibrosis, and cirrhosis [25]. In a recent study, the G allele of rs738409 in the PNPLA3 gene was shown to be a risk factor for NAFLD in children. The results, which determine the relationship between gene and polymorphism, showed that the risk of NASH with the GG genotype is higher than GC and CC genotypes [26]. According to Akkiz et al. study, the rs738409 C>G SNP is strongly associated with increased liver fat content and causes progression to NASH [27]. The adverse impact of SNPs in the PNPLA3 gene is well established in various populations. In a study on American, African, European, and Spanish subjects, rs738409 was powerfully associated with augmented liver fat and inflammation in all populations. In this study, the highest frequency of the G allele was found in the Spanish population [20]. Also, in another similar study, evidence of a strong association between the rs738409 variant and susceptibility to NASH was found in both Asian and Caucasian populations. However, this susceptibility was higher in the Caucasian population with the rs738409 variant [28]. Besides rs738409 C>G, there are some other PNPLA3 SNPs that may contribute to NAFLD incidence and/or progression. However, there are limited data in this regard. rs2294918 G>A, rs139051 T>C, and rs6006460 G>T are among the most important SNPs in PNPLA3 gene [29, 30]. Surprisingly some of these SNPs have protective role against rs738409 C>G and could potentially be utilized through gene editing techniques to target the expression of defective variants [31].

Hydroxysteroid 17-beta dehydrogenase 13 (HSD17B13) is another LD-associated protein with liver-specific function. This protein contributes to retinoid metabolism via retinol dehydrogenase (RDH) activity. HSD17B13 also contributes to the pathogenesis of NAFLD by targeting LDs in hepatocytes [32, 33]. Loss-of-function splice variant rs72613567 T>TA of the HSD17B13 gene diminishes the risk of NAFLD progression and chronic liver disease through regulation of lipid metabolism and decreasing liver LD biogenesis [34]. The A allele of rs72613567 is shown to have a protective effect against NAFLD, alcoholic liver disease, and hepatocellular carcinoma [35]. Notably, the presence of the HSD17B13 rs72613567 A allele mitigates the increase in alanine transaminase (ALT) and aspartate aminotransferase (AST) levels among carriers of PNPLA3 rs738409 G allele [36].

MBOAT7 is the other key gene in lipid metabolism, which encodes an enzyme with lysophosphatidylinositol acyltransferase activity that catalyzes phosphatidylinositol production. MBOAT7 causes lipid kinetics changes in liver cells by increasing phosphatidylcholines (PC) and phosphatidylserine contained arachidonic acid in the liver and reduces the risk of steatosis [37]. Recent studies have shown that MBOAT7-knocked-out mice suffer from TG and liver fat content increment [38]. The rs641738 C>T variant of MBOAT7 impairs lipid homeostasis by enhancing phosphatidylinositol turnover and promoting triglyceride synthesis, which consequently leads to liver steatosis and inflammation [38]. The increase in liver lipid content leads to an elevation in DNL and the progression of NASH through the overexpression of SREBP-1c, which functions as a transcription factor in the lipogenesis pathway [39]. In Lieu et al. study, the loss of function of the adjacent gene MBOAT7 boosts the progression of fatty liver disease. However, the effects of the rs641738 variant on the development of NAFLD seem to be distinguished in different ethnicities [40]. A cross-sectional cohort revealed that the rs641738 variant in the MBOAT7 gene is associated with an increased risk of NAFLD progression in European individuals [41]. Likewise, the rs626283 risk variant is demonstrated to influence the progression of NASH by modulating intrahepatic fat and affecting glucose metabolism in the Caucasian population [42].

### Lipid transport

An increase in fatty acid circulation enhances TG accumulation in the liver, which is the consequence of high fat uptake, an increase in adipose tissue lipolysis, and T2DM [43]. The imbalance between the uptake and export of TG in the liver is one of the main features of NAFLD. Lipids can either be released as VLDL particles or oxidized in mitochondria in order to be eliminated from the liver [44]. TM6SF2, a regulator of liver fat metabolism, prevents lipid aggregation by controlling TG secretion and hepatic LD content. According to a recent study, TM6SF2 silencing leads to reduced lipoprotein production and export, as well as developing small LDs in hepatocytes. Lacking the TM6SF2 gene dramatically increases ER stress and mitochondrial dysfunction in result of alterations in these organelles morphology [6]. Phosphatidylcholine is a major component of biological membranes which maintains the function of ER, membrane homeostasis and contact sites between ER and mitochondria [45]. The TM6SF2 gene deficiency reduces PC levels and results in shapeless ERs [6]. Although the precise function of TM6SF2 in the context of NAFLD is mostly unknown, a study by Li et al. demonstrated that overexpression of TM6SF2 reduced hepatic lipid accumulation in HFD-fed mouse models, whereas knockdown of TM6SF2 was shown to promote inflammation and hepatic lipid accumulation [12]. TM6SF2 rs58542926 increases the risk of lipid accumulation in hepatocytes and decreases circulating fatty acids in serum by reducing VLDL secretion [6]. Although the association of TM6SF2 rs58542926 and the spectrum of NAFLD disease is controversial, several studies have demonstrated that the loss-of-function variant rs58542926 could be considered a risk factor in NAFLD and fibrosis progression [46]. The TM6SF2 C>T variant downregulates the TM6SF2 protein expression and has been associated with decreased LDL levels and cardiovascular risk, as well as increased T2DM risk [12]. In a meta-analysis, Li et al. found that NAFLD risk increased with the presence of rs58542926 and demonstrated a positive correlation between rs58542926 and ALT in both children and adults*.* Moreover, this variant is negatively associated with total cholesterol, LDL, and TG [47]. A recent study in the Chinese Han population by Li et al. observed high levels of TG, AST, and ALT and showed an association between the TM6SF2 variant and NAFLD [48].

FA transportation into peripheral organs as TG-rich lipoproteins (VLDLs) is linked to ER stress and NAFLD progression. The assembly of VLDL accompanies by the function of apoB and MTTP genes, and low expression of hepatic MTTP is reported to associate with the pathogenesis of NAFLD [49]. Although several MTTP SNPs have been identified, a common polymorphism rs1800591-493 G>T contributes to NAFLD by decreasing the expression of MTTP and impairing the potential ability of this gene to export lipids [50]. Although Tan et al. found no association between NAFLD and the rs1800591 polymorphism of the MTTP gene in a meta-analysis, it is suggested that this polymorphism could be used as a biomarker for early diagnosis of NAFLD [51].

The gene APOC3 plays an essential role in the transport and clearance of residual chylomicrons. Aside from being involved in the formation of VLDL, APOC3 also functions as one of the major inhibitors of TG-rich particles [52]. The rs2854116 variant is associated with susceptibility to the development of NAFLD and IR by increasing the plasma concentration of Apoc3 and sequentially inhibiting the clearance of lipoprotein lipase and triglycerides. Consequently, the liver absorbs higher concentrations of chylomicron remnants leading to greater levels of TG accumulation [53]. A meta-analysis by Tong et al. reported that the APOC3 polymorphism rs2854116 might be involved in the development of NAFLD and could be a potential therapeutic target for NAFLD [54]. It has been shown that another mutation in APOC3 rs2070667 is responsible for exacerbating the pathological factors associated with NAFLD, mainly because of its inhibitory effect on PUFA-containing TG levels in serum [55].

FATP5, also called SLC27A5 is mainly expressed in the liver and participates in controlling FFA uptake. As well as carrying out its function as a fatty acid transporter, FATP5 can also activates long-chain fatty acids (LCFAs) through covalent coenzyme A attachment [56]. It has been reported that deletion or silencing of FATP5 reduces triglyceride levels in the liver and ameliorates diet-induced steatosis in rats [57]. The FATP5 variant rs56225452 gain-of-function was found to be associated with an increased risk of hepatic steatosis, elevated ALT levels, and enlarged insulin resistance [58].

Eventually, among the variants affecting liver damage through the metabolism of lipids, the most significant impact is related to the PNPLA3 I148M variant, followed by MBOAT7 rs641738 and E167K TM6SF2 [59].

### Glucose metabolism

**Fig. 2:**
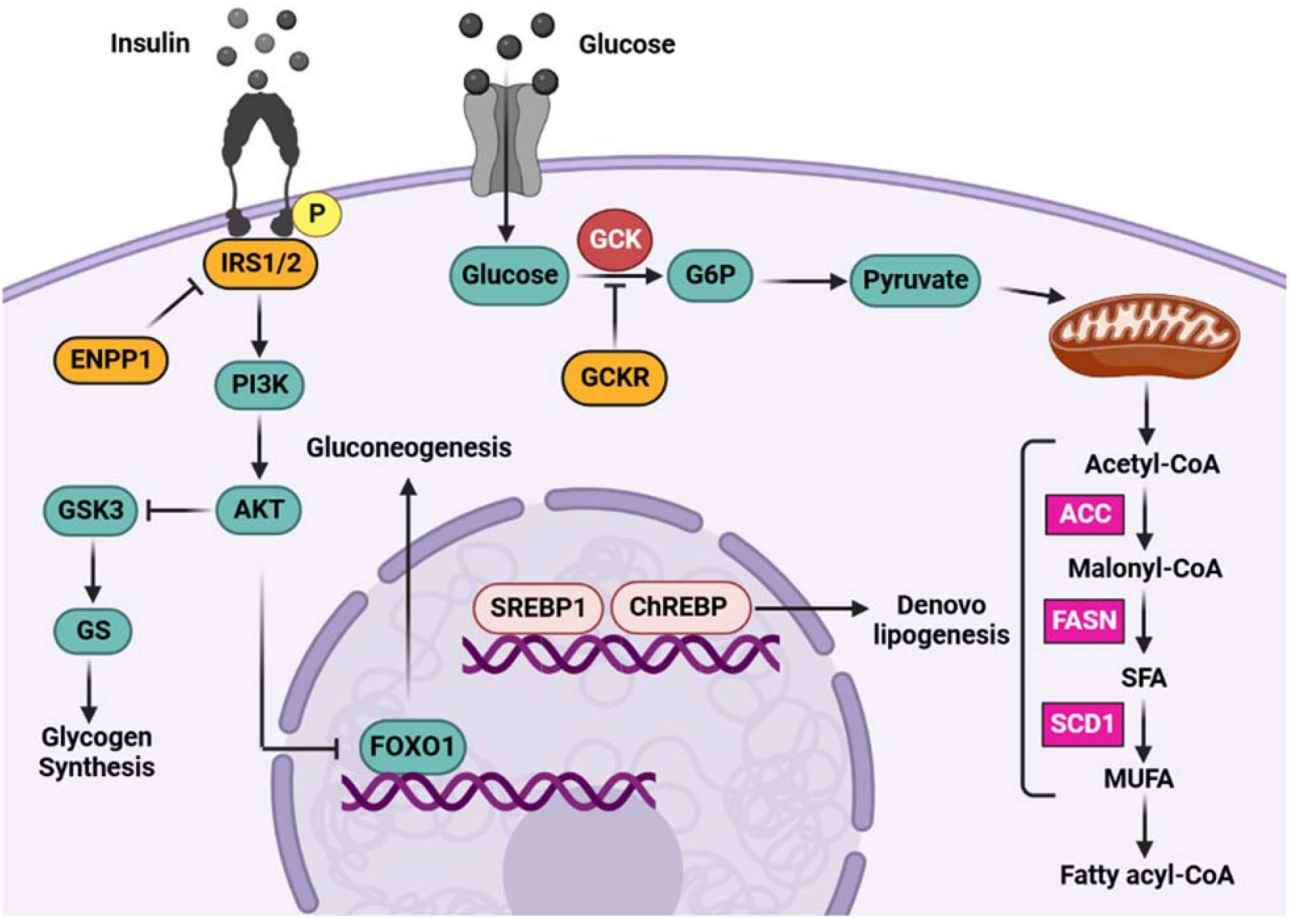
Schematic representation of the glucose–insulin signaling pathway. Binding of insulin to its receptor (IR) stimulates activation of downstream PI3K/Akt cascade. Activation of Akt by insulin results in glycogen synthesis and gluconeogenesis through GSK3 and FoxO1, respectively. ENPP1 interacts with Insulin receptor and inhibits its kinase activity. The GCK phosphorylates glucose in the hepatocyte to G6P and allows glucose to enter the cell. During glycolysis which is regulated by GCKR through inhibiting GCK, pyruvate is generated and transported to the mitochondria where it is decarboxylated to acetyl-CoA leading to de novo lipogenesis. The activity of SREBP1c is upregulated by insulin signaling, whereas ChREBP has been identified as a glucose-activated transcription factor, both of which contribute to de novo lipogenesis and fatty acid synthesis. During DNL, the ACC enzyme converts acetyl-CoA to malonyl-CoA, and then the FASN enzyme produces SFAs. Additionally, SCD1 transforms SFAs into MUFA, which is used as a substrate for the production of fatty acids. *FOXO1* Forkhead box protein O1, *PI3K* phosphatidyl inositol 3-kinase, *AKT* AKT serine/threonine kinase 1, *GS* glycogen synthase, *G6P* glucose-6-phosphate, *GCK* glucokinase, *GCKR* glucokinase regulator, *ChREBP* carbohydrate-response element-binding protein, *SREBP1* sterol regulatory element-binding protein 1, *FASN* fatty acid synthase, *SCD1* stearoyl-coa desaturase 1, *MUFA* monounsaturated fatty acids, *SFA* saturated fatty acid, *ACC* acetyl-CoA carboxylase

The liver is responsible for maintaining glucose homeostasis and insulin has a crucial function in this regard. Insulin controls glucose metabolism and results in glucose uptake through the phosphorylation of insulin receptor substrate, which regulates multiple downstream processes [74]. As Fig. 2 demonstrates, following the entrance of glucose in the liver, glucose 6-phosphate (G6P) is produced by glucokinase in several pathways, such as glycolysis and glycogenesis [75]. During glycolysis, the excess glucose in the liver is used to provide energy and can also turn into acetyl-CoA to synthesize free fatty acids. Moreover, extra glucose is stored as glycogen during glycogenesis, in which insulin activates GSK3/GS by the PI3K/AKT pathway [76].

Due to hyperinsulinemia in NAFLD, de novo lipogenesis and insulin resistance can be induced via upregulation of SREBP-1c and inhibition of insulin receptors through reduced expression and sensitivity of IRS1/2. Also, IR increases gluconeogenesis and diminishes hepatic glycogen synthesis, resulting in high glucose levels [77]. Consequently, ChREBP, a carbohydrate-signaling transcription factor, is activated and contributes to the progression of NAFLD by de novo lipogenesis. Type 2 diabetes mellitus (T2DM) can activate a similar mechanism in the liver. IR and high glucose, which are triggers of T2DM, accelerate DNL via SREBP and ChREBP cascades, respectively. Several studies have reported that T2DM patients are more likely to suffer from NAFLD [78]. There is growing evidence that multiple SNPs are associated with glucose metabolism dysregulation and IR, which are potential causes of liver dysfunction (Table 2).

**Table 2:** Genetic variants influencing NAFLD pathogenesis via glucose metabolism pathways

Gene	Function	Variant	Mutation type	Variant classification	Population	References
ALDOB	Fructose breaking down	rs1800546	Missense	Pathogenic	European	[91]
C2orf16	–	rs1919127	Missense	Benign	Korean	[92]
ENPP1	Insulin signaling inhibitor	rs1044498	Missense	Benign	Italian and British	[90]
GCK	Insulin release regulation	rs2041547	Intronic variant	Benign	Caucasians	[93]
GCKR	DNL regulation	rs1260326	Missense	Benign	Young Finns	[94]
		rs780094	Intronic variant	Benign	Indian	[95]
		rs149847328	Stop	Uncertain significance	Argentinian	[84]
IRS-1	Insulin signaling	rs1801278	Missense	Likely benign	Pakistan	[87]
IRS-2	Insulin signaling	rs2289046	3′ UTR variant	Benign	Iranian	[88]
PPP1R3B	Hepatic glycogen synthesis promoter	rs4240624	Intronic variant	Benign	American	[96]
PYGO1	Methylated histone binding activity	rs62021874	Intronic variant	Benign	European	[97]

*ALDOB* aldolase, fructose-bisphosphate B, *C2orf16* chromosome 2 open reading frame 16, *PPP1R3B* protein phosphatase 1 regulatory subunit 3B, *PYGO1* pygopus family PHD finger 1

The glucose accumulation in hepatocytes is controlled by the glucokinase enzyme encoded by GCK, which phosphorylates glucose to G6P during glycolysis. Subsequently, G6P is converted to pyruvate to generate acetyl-CoA, a substrate that participates in DNL to produce FFA and TG. Moreover, G6P can turn into glucose-1-phosphate and participate in the glycogen synthesis pathway. It is reported that GCK gene expression is upregulated in the fatty liver and has a significant correlation with liver triglyceride content and DNL-related genes, including FASN, ACC-1, and ACC-2 [79, 80]. Glucokinase regulator protein (GCKR), known as glucokinase inhibitor, is responsible for adjusting glucose storage and disposal, as well as controlling de novo lipogenesis through regulating glucose flow into hepatocytes [81]. GCKR rs1260326 (P446L) is a missense variant that increases glucose uptake and DNL by reducing the ability of glucokinase inhibitory effect [82]. The GCKR rs1260326-T is associated with metabolism-related mechanisms, such as glycolysis, fatty acid circulation, and saturation. Yuan et al. observed an increase in TG levels of rs1260326-T carriers and demonstrated an association between GCKR rs1260326-T and fatty liver by studying the elderly Chinese Han population [72]. Furthermore, Nahass et al. confirmed that GCKR rs1260326 allele T was associated with susceptibility to NAFLD [83]. Another GCKR gene variant, rs780094, is reported to increase triglyceride levels in the Chinese population [46]. Both GCKR variants of rs780094 and rs1260326 contribute to NAFLD, considering the activation of DNL [46]. Furthermore, a recent study of a rare nonsense mutation rs149847328 has demonstrated a decrease in GCKR protein expression in patients carrying the rs149847328 variant in comparison with NAFLD patients with the wild‐type allele [84].

To adjust glucose homeostasis in a normal physiological state, insulin binds to its receptor on hepatocytes and triggers the tyrosine kinase activity of the insulin receptor. Consequently, the IRS-1/2 is phosphorylated and activates the PI3K/AKT pathway, leading to gluconeogenesis suppression and glycogen synthesis through Forkhead box protein O1 (FOXO1) and GSK3, respectively. Furthermore, the activation of IRS can lead to an increase in SREBP1-C gene expression and a rise in fatty acid synthesis by promoting lipogenesis [85]. Thereby, malfunction of the IRS impairs insulin signaling and increases the risk of insulin resistance. The loss-of-function rs1801278 Gly927Arg polymorphism diminishes the IRS-1 activity and inhibits the insulin receptor autophosphorylation, causing a reduction in insulin signaling [86]. A recent Pakistani population study demonstrated a correlation between the Gly972Arg variant of IRS-1 and insulin resistance in T2DM [87]. IRS-2 polymorphisms are also linked to IR, T2DM, and hyperinsulinemia, in line with Dabiri et al. study that suggested a considerable association between the IRS-2 rs2289046 variant and NAFLD [88].

Another important gene in glucose metabolism is ENPP1, which encodes a transmembrane glycoprotein that inhibits insulin receptor activity and decreases insulin signaling. ENPP1 regulates insulin actions via physical interactions with the α-subunit and inhibiting the β-subunit of the insulin receptor. The overexpression of ENPP1 in the liver causes IR and declines glucose uptake [89]. Gain-of-function K121Q mutation promotes the interaction between ENPP1 and insulin receptors and consequently inhibits insulin signaling. Dongiovanni et al. demonstrated a correlation between the IRS-1 972Arg and ENPP1 121Gln with increased hepatic insulin resistance by measuring the AKT activity of patients suffering from fatty liver. In their study, patients who carried both ENPP1 and IRS-1 SNPs were more susceptible to developing fibrosis than those positive for ENPP1 or IRS-1. However, the role of the ENPP1 variant was more prominent in this regard [90].

## Internal cellular stresses and genetic susceptibility

Hyperglycemia and lipid accumulation in hepatocytes trigger cellular stresses mainly via disrupting ER function and mitochondrial damage [98, 99]. ER stress accompanies an overload of misfolded/unfolded proteins and gives rise to oxidative stress via enhancing reactive oxygen species (ROS) production. The ROS may activate different signaling pathways and cause genomic mutations and lesions in favor of NAFLD progression toward HCC [100, 101]. In addition to ER stress, elevated FFA β-oxidation in mitochondria also increases ROS production, which causes oxidative stress and mitochondrial damage [102, 103]. The defense mechanism of cells to reduce ROS is to induce mitophagy and remove the damaged mitochondria. However, the disruption in mitophagy during NAFLD enhances the inflammatory state, which plays a vital role in NAFLD progression toward steatohepatitis [104, 105].

The oxidative stress and ER stress contribute to LD accumulation in hepatocytes by activating crucial lipogenesis transcription factors, including SREBP-1c. To counteract the accumulation of lipid droplets, hepatocytes use lipophagy to degrade intracellular LDs [106, 107]. Accordingly, defective hepatic lipophagy is one of the key players in the progression of simple fatty liver to NASH [108]. It is demonstrated that genetic inhibition of lipophagy elevates TG and LD content, declines hepatic FA oxidation, and consequently induces NAFLD/NASH [109, 110]. Increased FFA β-oxidation in mitochondria leads to the production of high amounts of ROS [111]. In the normal physiological state, the increased ROS production in the mitochondria is negated by antioxidant enzymes such as superoxide dismutase (SOD), catalase (CAT), and glutathione peroxidase (GPX). The decline in the function of these enzymes and the subsequent elevation in ROS causes the disease to progress toward NASH [112].

**Fig. 3:**
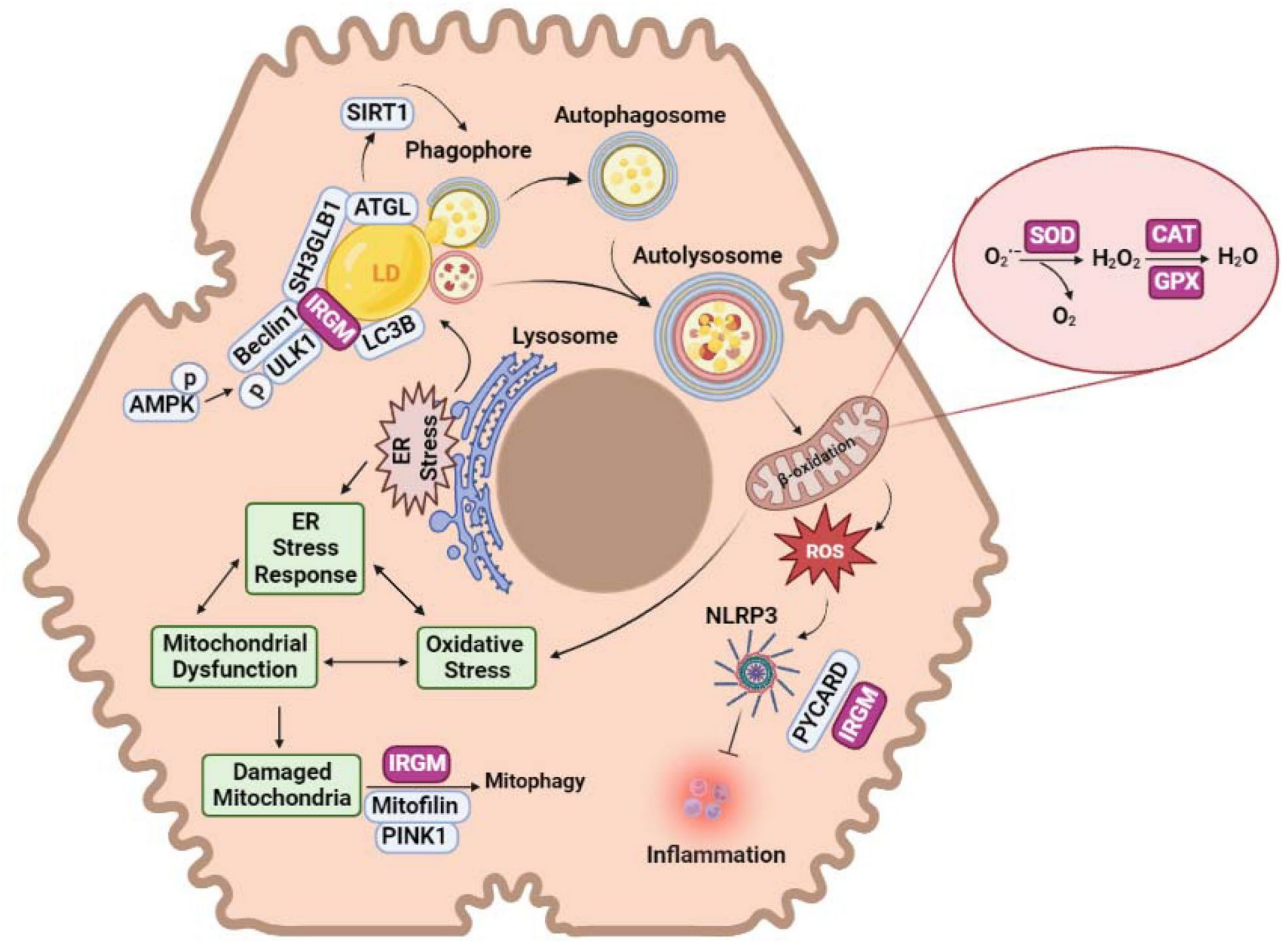
Schematic representation of cellular stresses in NAFLD. ER stress increases lipid droplet accumulation by activating factors involved in lipogenesis. IRGM plays an important role in increasing lipophagy of LDs. IRGM promotes lipophagy through complex formation with ULK1 and Beclin, binding to ATG16L1, and stimulation by Sirt1. Moreover, enhanced FFA β-oxidation in mitochondria by production of large amounts of ROS leads to a disruption of the electron transport chain. This defect leads to the leakage of e-, which immediately combines with oxygen to generate the superoxide anion radical, which is then converted to H_2_O_2_ by SOD2 activity. The antioxidant enzymes GPX1 and catalase convert H_2_O_2_ into H_2_O and O_2_. On the other hand, ER stress leads to mitochondrial damage by causing oxidative stress. IRGM regulates mitofilin stability during mitochondrial depolarization, leading to PINK1-Parkin-dependent ubiquitination and removal of defective mitochondria

As shown in Fig. 3, regulation of hepatic LD catabolism is conducted via lipophagy, in which the interaction of LDs with LC3 and adipose triglyceride lipase (ATGL) promotes SIRT1 activity [113]. In the initiation phase, immunity related GTPase M (IRGM) through phosphorylation of AMPK, ULK1, and Beclin-1, as well as cross-linking with ATG16L1 and SH3GLB1 causes lipophagy enhancement [114]. IRGM also controls mitophagy via regulating mitochondrial biogenesis and interacting with mitofilin and PINK1 [115]. Moreover, IRGM suppresses NF-κB and MAPK/p38 inflammatory pathways by inhibiting the activation of the NLRP3 and PYCARD complex [116]. A previous study demonstrated that IRGM expression is significantly lower in the liver of NAFLD patients [117]. In contrast, the overexpression of IRGM decreased lipid droplet content during NASH [118]. It was shown that the genetic defect in lipophagy mediated by IRGM rs13361189 and rs10065172 TT genotype interferes with mitochondrial function, disrupts liver fat metabolism, provokes inflammation, and induces hepatic steatosis (Table 3) [119]. In obese children and adolescents from a Han Chinese population, rs10065172 C>T has been identified as a polymorphism associated with NAFLD [120]. Also, in obese Italian children, the risk allele of rs10065172 is associated with increased plasma aminotransferase levels and mild steatosis [121].

**Table 3:** Genetic variants influencing NAFLD progression through cellular stress responses

Gene	Function	Variant	Mutation type	Variant classification	Population	References
IRGM	Modify hepatic lipophagy and mitophagy	rs10065172	Missense	Benign	Han Chinese	[120]
					Italian obese children	[121]
		rs13361189	Missense	Benign	American	[129]
SOD2	Protecting cells from oxidative stress	rs4880	Missense	Benign	Chinese	[112]
CAT	Detoxifies H_2_O_2_ in peroxisomes and mitochondria	rs1001179	Upstream variant	Benign	Chinese	[112]
GPX1	Reduce hydrogen peroxide to generate oxidized glutathione and water	rs1050450	Missense	Benign	Chinese	[112]
UCP3	Metabolism of superoxide radicals	rs3781907	Intronic variant	Benign	Brazilian	[130]
MTARC1	Metabolic processes in the liver	rs139321832	Stop	Likely Benign	American	[131]

*UCP3* uncoupling protein 3, *MTARC1* mitochondrial amidoxime reducing component 1

In the liver, SOD participates in lipid peroxidation, reduction of mitochondrial ROS, and protection against oxidative stress [122]. Total knockout or knockdown of the Sod2 gene causes ROS-derived disorders and lipid deposition. The expression of SOD2 is decreased in the liver of NASH patients [123]. CAT and GPX preserve the liver from lipid accumulation and inflammation by removing the hydrogen peroxide produced due to SOD2 activity [124]. Elevation of ROS increases lipid peroxidation, mitochondrial dysfunction, and apoptosis rate [125]. Thus, establishing a balance between oxidative stress and antioxidative factors protects cells from hepatic stellate cell stimulation and NASH induction [126, 127]. SOD2 rs4880 47T>C [128] and CAT-262C>T rs1001179 SNPs in the antioxidative genes disrupt their enzymatic activity (Table 3). SOD2 rs4880 47T>C variant is associated with advanced fibrosis in an allele dosage-dependent manner [112]. Concludingly, ROS content increases and enhances the susceptibility for developing NASH and advanced fibrosis in the carriers of these SNPs (Table 3).

## Genetic factors related to immune system imbalance

Inflammation and immune responses caused by cellular stress and cellular damage are the main causes of the progression of steatosis toward NASH [132]. Inflammation is characterized by immune activation through various signaling pathways, lipid accumulation, and oxidative stress [133]. However, NASH is not only associated with metabolic risk factors, but also with genetic alterations. Accordingly, polymorphisms in genes encoding inflammatory cytokines could lead to some liver disease. The most significant associations are brought by genetic variants involved in the regulation of inflammation, such as IL-32, TNF-α, IL-6, and IL-1β, which are frequently altered in NAFLD/NASH (Table 4).

**Table 4:** Genetic variants associated with NAFLD progression by inflammatory signaling pathways

Gene	Function	Variant	Mutation type	Variant classification	Population	References
CNR2	Inflammation signaling regulator	rs35761398	Missense	Uncertain significance	Italian children	[154]
TNF-α	Proinflammatory cytokines; induces neutrophils to develop pyroptosis	rs1800629	Upstream variant	Benign	Iranian	[155]
IL-1β		rs16944	Upstream variant	Benign	Caucasian	[149]
IL-6		rs1800795	Upstream variant	Benign	Caucasian	[149]
IL-27	Central coordinator of Treg cell effector, functions during inflammation	rs4788084	Upstream variant	Benign	Indian	[63]
		rs2275913	Upstream variant	Benign	Turkish children	[156]
IL-13	Upregulates the expression of collagens and other pro-fibrotic genes	rs20541	Missense	Likely Benign	Caucasian	[157]
STAT6	PPARγ action modulator	rs167769	Intronic variant	Benign	Caucasian	[157]
IL-28B	Immune response	rs12979860	Intronic variant	Benign	Italian	[158]

*IL-27* interleukin 27, *IL-13* interleukin 13, *STAT6* signal transducer and activator of transcription 6, *IL-28B* interleukin 28B

**Fig. 4:**
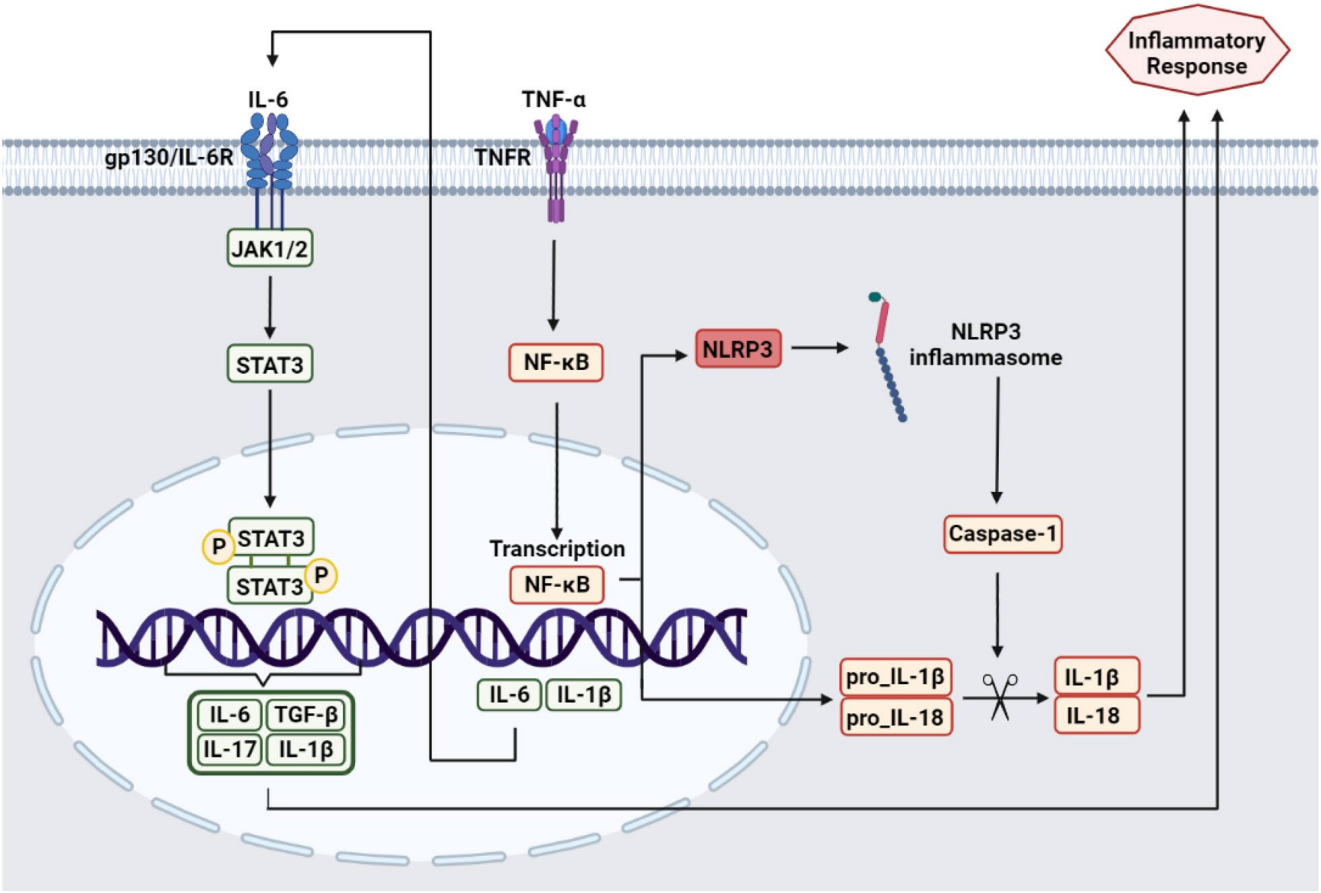
Schematic representation of the relationship between inflammation and NASH disease progression. Upon stimulation with TNF-α, the NF-κB pathway is activated, as indicated by phosphorylation and nuclear translocation of NF-κB and transcription of its target genes, including IL-6 and IL-1β. Newly synthesized IL-6 is secreted by cells and binds to the IL-6R in an autocrine or paracrine manner, leading to activation of the IL-6R/gp130 complex and intracellular JAK1/2 kinases. STAT3 proteins are then phosphorylated by JAK1/2, dimerize, enter the nucleus, and initiate transcription of STAT3-dependent genes such as TGF-β, IL-6, IL-17, and IL-1β, which contribute to the development of inflammatory responses Alternatively, the first signal for NLRP3 inflammasome activation is NF-κB-mediated NLRP3 transcription. When activated, the NLRP3 inflammasome converts caspase-1, pro-IL-18, and pro-IL-1β into active forms, triggering an inflammatory response. *IL-6R* interleukin-6 receptor, *gp130* glycoprotein 130

IL-32, as a pro-inflammatory cytokine, is highly expressed in the liver during liver disease [134]. Its transcription is upregulated in obese individuals with severe NAFLD (particularly in carriers of the PNPLA3 I148M risk variant) and can be induced by lipotoxicity in hepatocytes [135]. A recent study reported that IL-32 rs9788910 is associated with elevated liver enzyme levels and NAFLD progression [136]. IL-32 elevates the localization of STAT3 in the IL-6 promoter through STAT3 phosphorylation. Notably, the hepatic STAT3 signaling is increased in patients carrying the PNPLA3 risk variant [137–139]. IL-32 may also affect the course of NASH progression by enhancing the expression of IL-1, TNF-α and IL-8 through the NF-κB and the p38/MAPK pathways [140]. In fact, when NF-κB is activated by TNF-α stimulation, the expression of NLRP3 and pro-IL-1β increases (Fig. 4). NLRP3 is a critical component of innate immunity, highly expressed in the Kupffer cells [141]. Increased expression of NLRP3 has a substantial role in obesity-induced inflammation and worsens NASH [142]. NLRP3 inflammasome complex formation causes caspase 1-dependent release of the pro-inflammatory cytokines, including IL-1β and IL-18 [143]. IL-18 triggers the secretion of other pro-inflammatory cytokines, such as TNF-α, IL-1β, and IL-8. The cytokines produced in the liver by Kupffer cells and hepatocytes negatively affect lipid metabolism and hepatic inflammation [144]. Therefore, it is not surprising that higher serum TNF-α levels were found in NASH patients compared to healthy controls. In the Russian population, the association of TNF gene polymorphism -308G>A rs1800629 with the development of NASH has been determined. Carriers of the A allele of the TNF gene marker -308G>A significantly increase the risk of developing NASH [145].

IL-6 is another crucial factor in inflammation, acting mainly through the IL-6/STAT3 pathway [146]. The IL-6-174G>C rs1800795 is associated with NASH progression and can also determine the genetic predisposition to develop this disease [147]. It also increases the production of various inflammatory cytokines through the synergistic interaction between STAT3, followed by the hyper-activation of NF-κB [148]. In a study on Caucasian NAFLD patients, the presence of the IL-6 rs1800795 C allele accompanied an escalated risk for severe steatosis, whereas it was associated with less IL-6 expression in the liver and lower progressive inflammation and fibrosis [149]. Conversely, patients with the G allele of rs1800795 have boosted risks for the progression of liver diseases, especially NASH and ALD, toward severe forms of the disease [150]. According to recent studies, IL-6 has modulated activation of the JAK–STAT3 signaling pathway through cooperation with the inflammatory cytokine IL-1β (Fig. 4). Indeed, IL-1β in NAFLD contribute to steatosis’ progression toward NASH and fibrosis. In addition, IL-1β also plays an important role in stimulating the activation of hepatic stellate cells and the accumulation of triglycerides and cholesterol in hepatocytes and the formation of fat droplets. In this regard, a Japanese cohort revealed that the gain-of-function of the IL-1β-511C>T rs16944 variant plays a major role in the development of NAFLD through its involvement in disease stages ranging from simple steatosis’ progression toward NASH and fibrosis [149, 151].

On the other hand, the known effects of the CB2 receptor in modulating the inflammatory response by inhibiting NF-κB transmission into the nucleus, reducing the production of TNFα and IL-6, IL-1B and increasing IL-10 play an important role in preventing the progression of steatosis to steatohepatitis [152, 153]. CNR2 is associated with high expression in the liver and a missense mutation of this receptor rs35761398 is demonstrated to obliterate the anti-inflammatory receptor function, induce an inflammatory state, and increase the susceptibility for NASH [154].

### Genetic predisposition in lean NAFLD individuals

Despite the critical role of obesity in NAFLD induction and progression, about 5% to 26% of patients who express this condition have a normal body mass index (BMI). These patients are categorized as “lean NAFLD” [159]. Surprisingly, a recent systematic review indicated that lean NAFLD accompanies worse outcomes and higher mortality rates compared to NAFLD in obese patients. A more prominent presence of underlying genetic disorders, as well as inefficiencies with lean NAFLD diagnosis and management, may explain these findings [160].

Lean NAFLD has a distinct distribution all over the world. Its prevalence in Asians is higher compared to the people of western countries, even though stricter cutoffs define them (BMI < 23 kg/m^2^ for Asians vs. BMI < 25 kg/m^2^ for non-Asians) [161, 162]. The epidemiological findings further imply the pivotal role of the genetic component in the pathophysiology of lean NAFLD [163].

Lean NAFLD is categorized into two principal subtypes. The first subtype constitutes the majority. It comprises metabolically obese normal-weight patients, who usually have increased visceral adiposity and waist circumference and develop insulin resistance [162]. Many of these patients have hepatic steatosis and exhibit a lipodystrophic phenotype due to an impaired ability to store lipids subcutaneously [164]. Lean NAFLD individuals of the first subtype and obese patients with NAFLD have shared pathophysiology, and they identically respond to lifestyle interventions [160].

However, the second subtype is substantially caused by defective genetic disorders. Population-based studies on lean individuals have identified several SNPs contributing to NAFLD induction and progression. NAFLD induction in the studies is usually defined as the presence of intrahepatic triglyceride levels above 5%. Among them, SNPs in PNPLA3, TM6SF2, APOB, MTTP, PEMT, and CETP are the most established, while the contribution of other genetic variants has not been confirmed [69, 70, 165, 166].

PNPLA3 rs738409 is the most commonly studied genetic variation in the context of lean NAFLD induction and progression. Recent studies have revealed that the PNPLA3 rs738409 G allele is more prevalent among lean NAFLD individuals than in obese patients and it escalated the risk of NAFLD incidence over twice in these patients [167–169]. On the other hand, the PNPLA3 G allele pathogenic contribution is not restricted to NAFLD induction. In a retrospective cohort study conducted on biopsy-proven Italian NAFLD patients, Fracanzani et al. demonstrated that the presence of the rs738409 G allele was the only variable that could enhance the risk of NASH and liver fibrosis development [170].

As previously discussed, TM6SF2 rs58542926 C>T is indicated to contribute to NAFLD induction and its progression to advanced fibrosis [171]. Intriguingly, there is evidence to demonstrate a higher prevalence of this risk variant in lean NAFLD patients [167, 170, 172]. Cohort and cross-sectional studies on biopsy-proven NAFLD patients demonstrated that the risk variants of the TM6SF2 also increase the susceptibility to inflammation, NASH, and fibrosis to a greater proportion in lean individuals compared to obese subjects [170, 173].

More than 60 different loss-of-function mutations could occur in the APOB gene and cause hypobetalipoproteinemia. This autosomal codominant disorder hampers the production of functional APOB proteins, which per se impedes VLDL secretion and leads to hepatic accumulation of TGs. Since hypobetalipoproteinemia accompanies fat malabsorption and failure to thrive, NAFLD screening should be considered in the affected lean subjects [174]. Notably, mutations in the MTTP gene, which encodes the apoB chaperone protein, can also inhibit the production of beta-lipoproteins and induce a more severe form of the disorder called abetalipoproteinemia [175]. To evaluate the effect of APOB and MTTP genetic polymorphisms on lean NAFLD, Di Filippo et al. conducted a cohort study and merged their results with data derived from previously published works to add statistical strength to their research. They observed that in spite of the mutations in the APOB gene, mutations in the MTTP gene are associated with significantly lower BMI in patients (mean BMI of 25.3 and 19.7, respectively), indicating the close correlation between MTTP polymorphism and lean NAFLD [166].

The PEMT gene encodes enzymes contributing to the hepatic synthesis of phosphatidylcholines. rs7946 C>T in PEMT is demonstrated to simultaneously protect against obesity and insulin resistance while exacerbating NAFLD severity in animal studies [176, 177]. A study on the PEMT mRNA expression in NAFLD patients further supported these findings and reported a significant correlation between lower PEMT mRNA levels (due to missense mutation) and lower BMI and NASH incidence [177]. A clinical study using the whole-exome sequencing method on lean NAFLD-inducing genetic variants further confirmed previous findings and demonstrated a threefold higher NAFLD incidence in lean subjects with the defective variant [70].

CETP has a critical role in transferring triglycerides between lipoproteins. The association of two SNPs in the CETP gene (rs12447924 and rs12597002) and NAFLD development in lean subjects have been documented in a cohort study of Australian adolescents. In this study, the prevalence of NAFLD was 3–5% in lean wild-type females. However, the risk for lean NAFLD was significantly higher among female homozygotes (25–33%) and heterozygotes carriers of SNPs (10–15%). Surprisingly, a similar association was not recorded in male or obese subjects, necessitating further in-depth studies to uncover underlying etiologies [69].

Collectively, lean individuals should not be considered privileged from NAFLD-related severe complications. Especially those harboring high-risk genetic variants might be subjected to unexpected health complications, necessitating effective diagnostic and therapeutic measures for these vulnerable subpopulations.

## Application of the genetic knowledge in precision medicine of NAFLD

Although to date there is no clinically available precision treatment for NAFLD tackling a special genetic variant, the genetic background consideration for the treatment of each patient substantially determines their response to available treatments [178]. Genetic data could be applied to intervene in the disease course in several ways. Primarily, it can help reduce the disease burden by proposing behavioral modifications to each patient. As so, a cohort study revealed that a 12-month lifestyle modification accompanied a more pronounced decrease in hepatic fat content, total blood cholesterol, and LDL in the carriers of the PNPLA3 I148M (especially in homozygous carriers) compared to wild-type individuals [179].

Secondarily, genetics could aid us in the early diagnosis and stratification of patients at high risk for developing severe forms of NAFLD. Although low-risk populations only require interventions after appearing the clinical manifestations of the disease, those at higher risk of NAFLD progression necessitate more invasive diagnostic procedures (i.e., liver biopsy) and early therapeutic interventions [15]. Notably, utilizing genetic information for precision cancer screening is a promising approach, which previously has shown efficacy in predicting the incidence of various cancers. SNPs in genes such as PNPLA3, GCKR, TM6SF2, and MBOAT7 are independently associated with hepatocarcinogenesis [180]. However, due to the complexity of contributing factors in NAFLD progression, each SNP is unexpected to be a strong risk predictor, and guidelines do not advocate routine genotyping to find them [7, 181]. Thus, to establish accurate HCC risk estimation models, besides the genetic profile of each individual, the condition of other HCC risk factors, such as diabetes and obesity, should be considered [180]. On the other hand, some genetic variants have inhibitory/inducive roles in the pathogenesis of other diseases, which may affect the screening strategies. For instance, TM6SF2 risk variant carriers are less likely to develop cardiovascular disease and require a lower threshold for cardiac disease screening. In line with the increasing demand for NAFLD precision medicine, various companies have recently emerged which introduce risk score assessment services easily available for each individual [7].

Ultimately, pharmacotherapy could be personalized according to genetic data. PNPLA3, TM6SF2, HSD17B13, GCKR, and DGAT2 genetic variations have gained much interest in the genetic-based precision medicine of NAFLD (Table 5). Due to the strongly implemented role of the PNPLA3 I148M variant in NAFLD induction and progression, the primary focus of recent research is on this genetic polymorphism [178]. PNPLA3 high-risk populations are demonstrated to not benefit from conventional therapeutic options targeting hepatic lipogenesis [14]. A multi-center cohort study on NAFLD patients showed that the protective effect of statin therapy, as a means to inhibit cholesterol synthesis, on steatohepatitis was significantly lower in the carriers of the PNPLA3 risk variant. In contrast, in this study, carriers of the TM6SF2 risk variant and wild-type individuals benefited from statin therapy [182]. Likewise, Omega-3 reduces the expression of SREBP1c (a regulator of hepatic lipogenesis) and consequently suppresses de novo lipogenesis. A randomized controlled clinical trial (registration number NCT00760513) evaluating the effect of omega-3 on NAFLD treatment revealed that in spite of TM6SF2 risk variant carriers, those harboring PNPLA3 148M were less responsive regarding the reduction in hepatic fat content. It might be because of already existing reduced de novo lipogenesis in PNPLA3 I148M carriers [183]. Overcoming undesirable PNPLA3 phenotype could be achieved via three major approaches, including targeting PNPLA3 with (i) RNA interference, (ii) small molecules, or (iii) interfering NAFLD-related metabolic pathways [184].

**Table 5:** Clinical trial studies targeting NAFLD-related gene variants

Targeted gene	Drug name	Mechanisms	Stage of development	Outcome	References
PNPLA3	ION839	RNA interference using antisense oligonucleotides	Phase 1	Completed	[184] NCT04142424
				Recruiting	[184] NCT04483947
HSD17B13	ARO-HSD	Post-transcriptional gene silencing using RNAi	Phase 1	Decreased HSD17B13 mRNA Decreased HSD17B13 protein Decreased AST Decreased ALT	[189, 194] NCT04202354
HSD17B13	ALN-HSD	Post-transcriptional gene silencing using RNAi	Phase 1	Active, not recruiting	NCT04565717
HSD17B13	ALN-HSD	Post-transcriptional gene silencing using RNAi	Phase 2	Recruiting	NCT05519475
HSD17B13	AZD7503	RNA interference using antisense oligonucleotides	Phase 1	Active, not recruiting	NCT05560607
DGAT2	ION224	RNA interference using antisense oligonucleotides	Phase 2	Active, not recruiting	NCT04932512

RNA interference using antisense oligonucleotides (ASOs) is a novel strategy, targeting the mRNA to reach long-lasting downregulation of the PNPLA3 proteins translation in the carriers of risk variants [184]. Concordantly, Linden et al. conducted a preclinical study on PNPLA3 148M harboring mice fed a NASH-inducing diet. They utilized ASOs to downregulate the production of PNPLA3 mutant proteins and demonstrated a significant reduction in liver fat content, inflammation, and fibrogenesis [185]. Based on such promising outcomes, an ASO compound, namely ION839, is registered for phase 1 clinical trials in obese NASH subjects homozygous for the PNPLA3 risk variant (NCT04142424, NCT04483947).

Another potential approach is based on using small molecules to negate the detrimental effects related to the PNPLA3 I148M variant. Schwartz et al. have recently demonstrated the ability of an anti-cancer small molecule called momelotinib to suppress PNPLA3 expression in human hepatocytes and stellate cells via inhibiting the BMP/ACVR1/SMAD signaling pathway [186].

Interfering metabolic pathways contributing to undesirable effects related to the PNPLA3 I148M variant (such as HSD17B13 inhibition) is another promising approach to counteract the NAFLD burden [139]. PNPLA3 G allele carriers in NAFLD patients exhibit more severe forms of the disease, and the prevalence of advanced liver fibrosis, cirrhosis, and HCC is higher among them. Notably, simultaneous carriage of the HSD17B13 rs72613567:TA variant is indicated to negate the detrimental effects related to the presence of the PNPLA3 148M alleles [187]. Using exome sequence data from over 46 thousand individuals, Abul-Husn et al. demonstrated the capability of the HSD17B13 splicing variant (rs72613567:TA) in dampening the mRNA expression of PNPLA3 and its related liver injury in an allele dosage-dependent manner. Accordingly, they proposed HSD17B13 inhibition as a potential strategy to modify the risk of NAFLD progression in PNPLA3 148M allele carriers [35]. Concordantly, another study indicated that the HSD17B13 rs72613567:TA presence significantly attenuates the risk of alcohol-induced cirrhosis and HCC in the PNPLA3 148M allele carriers [188]. Due to the proven role of the rs72613567 insertion/deletion variant of HSD17B13, it could serve as a potential target for genetic-based precision medicine to treat NASH and liver fibrosis [15]. The application of post-transcriptional gene silencing using RNAi to suppress HSD17B13 expression has recently been introduced by Arrowhead (NCT04202354) and Alnylam Pharmaceuticals (NCT04565717) as a potential solution to treat NAFLD/NASH. Data released from phase I of the Arrowhead clinical trial indicated promising results regarding suppression of HSD17B13 at mRNA and protein levels as well as serum ALT and AST of patients. Likewise, INI-678 (an HSD17B13 inhibitor), introduced by Inipharm, has demonstrated efficacy in decreasing liver fibrosis in a human liver-on-a-chip-model, opening a gate for broader application of small-molecule therapy in genetic-based precision medicine of NAFLD [189]. Interestingly, other members of the HSD17B13 family, such as HSD17B11, with high similarity and widely recognized binding sites for small molecules, could serve as substitutes to improve the number of available choices [15].

However, targeting the TM6SF2 gene directly may not be a proper idea in NAFLD-based precision medicine. Although the upregulation of the TM6SF2 gene leads to a decrease in NAFLD incidence, it accompanies an unwanted increase in the content of blood lipids. It enhances the risk of cardiovascular diseases such as myocardial infarction [190]. Conversely, de novo lipogenesis targeting is one of the most effective NAFLD therapies for TM6SF2 and GCKR risk variant carriers [178]. In this context, acetyl-CoA carboxylase (ACC) inhibitors and fatty acid synthase (FAS) inhibitors, which target critical enzymes in the de novo lipogenesis process, have gained much interest [191]. TM6SF2 risk variant accompanies enhanced de novo lipogenesis and decreased VLDL secretion capability. An ACC inhibitor called MK-4074 has recently shown promising results regarding de novo lipogenesis decrease and prevention from liver steatosis and NASH in the carriers of the TM6SF2 defective variant [12]. Likewise, the GCKR P446L variant is associated with elevated glucokinase activity, glycolysis, hepatic glucose uptake, and de novo lipogenesis. Accordingly, it could be hypothesized that ACC and FAS inhibitors can be effective choices for treating GCKR P446L carriers. However, further studies should be conducted to corroborate the idea [181].

NAFLD genetic risk variants could also predict the liver-correlated adverse effects of other drugs. PNPLA3 and TM6SF2 risk variant carriers are indicated to enhance the risk of liver damage in response to some anti-diabetic agents, while some other anti-diabetic medications privileged these events. To hit on an example, PNPLA3 risk carriers are indicated to develop more liver fat accumulation as well as AST and ALT enzyme elevation following the treatment with basal insulin peglispro compared to insulin glargine [192, 193].

## Conclusion

Several polymorphisms are known to be associated with the pathogenesis of NAFLD and its progression to advanced stages. Accordingly, various molecular mechanisms might be affected, including lipid metabolism and transport, glucose metabolism, oxidative stress, ER stress, and inflammation. Gene polymorphisms could explain patient variability in response to treatment and the rate of disease progression. To date, polymorphisms in PNPLA3, TM6SF2, HSD17B13, MBOAT7, and GCKR have attracted more attention in the use of disease-associated variants for precision medicine. However, there is an urgent need for further research to explain the precise molecular mechanisms of these SNPs and pave the way for the development of new drugs. In addition, the possibility that genetic variations may vary by population group and ethnicity must also be considered. Therefore, future studies are needed to investigate other variants that may be associated with NAFLD pathogenesis, intending to screen patients and personalize the treatments.

## Data Availability

Not applicable.

## Abbreviations

NAFLD: Non-alcoholic fatty liver disease
NASH: Non-alcoholic steatohepatitis
HCC: Hepatocellular carcinoma
T2DM: Type two diabetes mellitus
SNP: Single nucleotide polymorphisms
DNL: De novo lipogenesis
IR: Insulin resistance
ER stress: Endoplasmic reticulum stress
GWAS: Genome-wide association studies
LD: Lipid droplet
FFA: Free fatty acid
HSC: Hepatic stellate cells
PC: Phosphatidylcholine
LCFA: Long-chain fatty acid
ROS: Reactive oxygen species
BMI: Body mass index
ASO: Antisense oligonucleotide
